# Recommendations for nutritional supplements for dry eye disease: current advances

**DOI:** 10.3389/fphar.2024.1388787

**Published:** 2024-05-30

**Authors:** Ying Cong, Yibing Zhang, Yutong Han, Yunlong Wu, Dan Wang, Bingjie Zhang

**Affiliations:** Department of Ophthalmology, The First Hospital of Jilin University, Changchun, China

**Keywords:** oxidative stress, L-carnitine, lactoferrin, probiotics, coenzyme Q10, spermidine, royal jelly

## Abstract

Dry eye disease (DED) represents a prevalent ocular surface disease. The development of effective nutritional management strategies for DED is crucial due to its association with various factors such as inflammation, oxidative stress, deficiencies in polyunsaturated fatty acids (PUFAs), imbalanced PUFA ratios, and vitamin insufficiencies. Extensive research has explored the impact of oral nutritional supplements, varying in composition and dosage, on the symptoms of DED. The main components of these supplements include fish oils (Omega-3 fatty acids), vitamins, trace elements, and phytochemical extracts. Beyond these well-known nutrients, it is necessary to explore whether novel nutrients might contribute to more effective DED management. This review provides a comprehensive update on the therapeutic potential of nutrients and presents new perspectives for combination supplements in DED treatment.

## Graphical Abstract

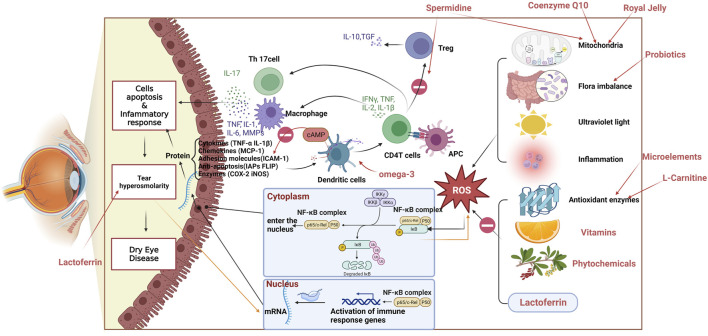

## 1 Introduction

**FIGURE 1:**
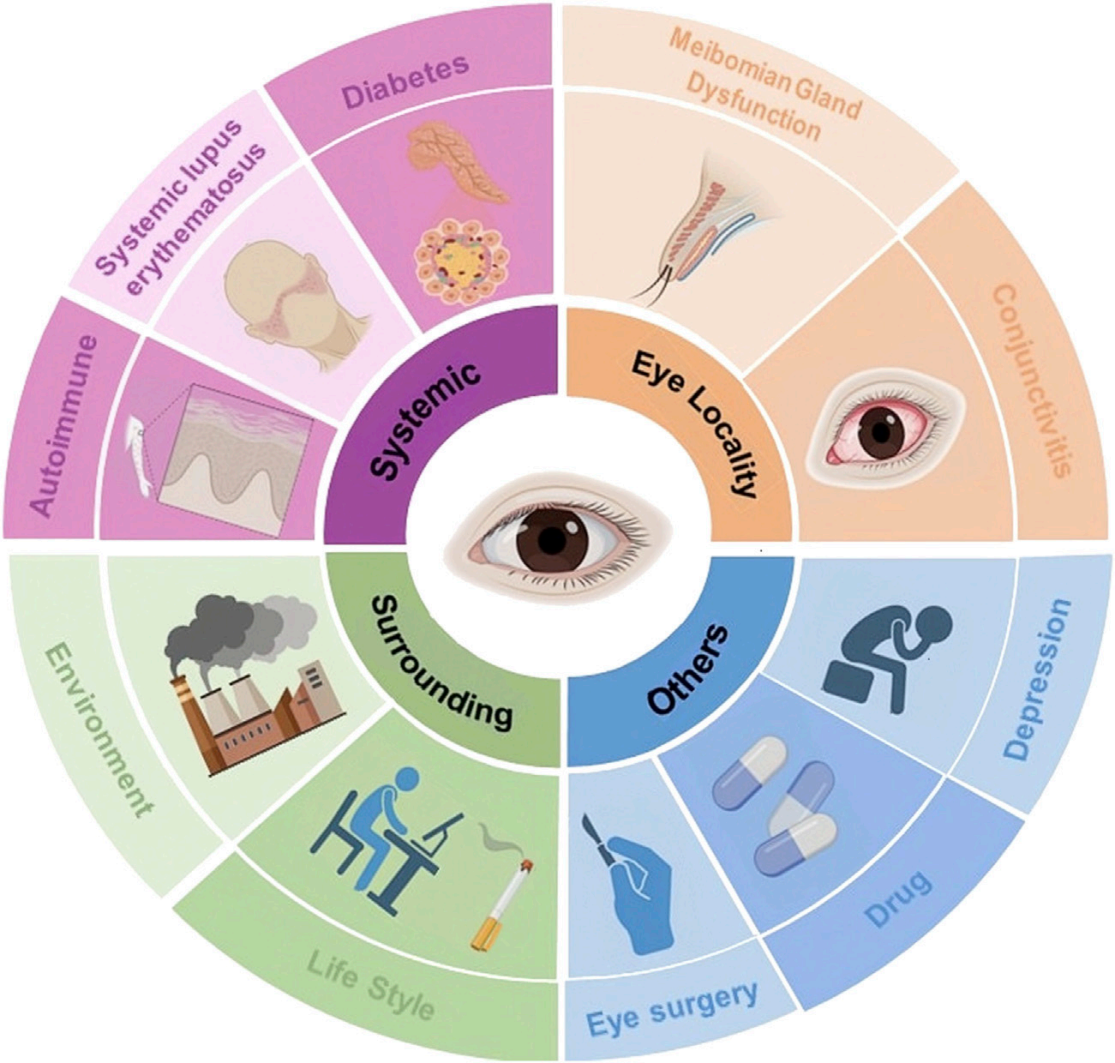
Classification of etiologic and risk factors for dry eye disease.

Dry Eye Disease (DED) is a complex, chronic ocular surface disease arising from diverse etiologies, significantly impacting quality of life (Figure 1) (Liu et al., 2022; Coassin et al., 2023; Craig et al., 2023; Kam et al., 2023; Montero-Iruzubieta et al., 2023; Narang et al., 2023; Buonfiglio et al., 2024; Xiong et al., 2024). The ocular surface system consists primarily of the cornea, conjunctiva, lacrimal glands, and meibomian glands, collaboratively maintains a smooth refractive surface and protects the visual system. These components produce the tear film’s three layers: lipid, aqueous, and mucin, which are crucial for ocular lubrication and protection (Gipson, 2007). Any disruption in these structures can lead to instability or imbalance in the ocular surface system, resulting in ocular discomfort symptoms and visual disturbances (The definition, 2007; Craig et al., 2017). DED primarily manifests in two subtypes: low tear production and excessive evaporation. Both subtypes lead to high osmotic pressure, inflammation, and damage to the epithelial cells. This creates a vicious cycle of DED (Martinez-Carrasco and Fini, 2023).

The prevalence of DED, which surged to as high as 61.0% during the COVID-19 pandemic (Ji et al., 2023), underscores the influence of increased exposure to visual display terminals and widespread mask usage during the pandemic, which likely exacerbated the condition by decreasing blink frequency and increasing thermal air currents over the ocular area, thus enhancing tear evaporation rates (Uwimana et al., 2022; Schargus et al., 2023). Early detection and management of DED are imperative to reduce both economic and psychological impacts and to maintain visual function (Mylona et al., 2023; Zhmud et al., 2023). Diagnosis needs to be combined with patient history, symptom assessment (Ocular Surface Disease Index), and auxiliary examination [tear film stability: break-up time (BUT) test and tear secretion: Schirmer’s test] (Schiffman et al., 2000; Jacobi and Cursiefen, 2010; Humayun et al., 2024) to conduct a comprehensive assessment.

At present, the treatment of DED is recommended to target the etiology. For meibomian gland dysfunction, intense pulsed light therapy is advocated to reduce inflammatory mediator release and clear gland blockages (Vigo et al., 2019). Additionally, when the aqueous layer is compromised, the use of artificial tear drops and secretagogues, such as diquafosol sodium eye drops, is recommended to maintain ocular surface lubrication and dilute inflammatory cytokine concentrations. The use of tear point plugs can also diminish the need for frequent application of eye drops in severe cases of DED. Pharmaceutical agents that enhance membrane-bound mucin and mucin secretion are employed to augment the mucin layer. Furthermore, treatments targeting the epithelium include autologous serum drops and Rebamipide ophthalmic suspension (Kojima et al., 2020). Immunosuppressants and low-dose steroid drops have also been recommended as anti-inflammatory treatments (Messmer, 2015; McCann et al., 2024). However, it is important to note that the extended use of topical anti-inflammatory agents and corticosteroids is limited due to their potential long-term adverse effects, such as cataracts and steroid-induced glaucoma (Teo et al., 2023; Wykrota et al., 2023).

Recent research highlights the significant roles of nutritional deficiencies (Castrejón-Morales et al., 2020; Chakraborty and Chandra, 2021; Jain et al., 2022; Markoulli et al., 2023), inflammation (Wu et al., 2024), and oxidative stress (Li et al., 2022a) in DED’s pathogenesis. With the rising awareness of oxidative stress and its impact on visual function, many Researchers are exploring the potential benefits of antioxidant therapy through nutrient supplementation. Numerous studies have demonstrated the value of nutrients, including Omega-3 fatty acids (ω-3FAs), vitamins, microelements, and phytochemicals, in preventing and treating DED (Iddir et al., 2020). This review provides an overview of effective nutrients in the management of DED, highlighting rising beneficial nutrients such as L-carnitine, lactoferrin, probiotics, spermidine, coenzyme Q10, and royal jelly. We summarize the sources, primary functions, and recommended dietary intakes for adults across ten categories (23 subcategories) of nutrients included in this review (Table 1). While these nutrients are available from food sources, patients with imbalanced diets or those experiencing discomfort symptoms may opt for commercially available nutritional supplements. However, it is crucial to evaluate the long-term safety of these nutrients, as extensive research indicates that nutrient supplementation can pose risks, including gastrointestinal distress, an increased incidence of bleeding, and a heightened prevalence of cancer and certain diseases, particularly in immunocompromised individuals and smokers.

**TABLE 1:** Sources, functions, and recommended nutrient intake (RNI) of nutrients related to eye health.

Nutrient	Sources	Main functions	RNI for adults
L-carnitine	Lean meat, liver, heart, yeast, lamb, milk, avocado, kiwifruit, grape, papaya	Facilitates cellular productivity, delivery of fatty acids for their metabolic use, metabolism of eye muscle tissue, and antioxidant	——
Lactoferrin	Humans, cattle, etc., secrete milk	Antibacterial, antiviral, immune-modulatory, and anti-inflammatory	——
Probiotics	Yogurt, kimchi, natto, cheese	Supports immune function and digestive tract health	——
Coenzyme Q10	Meat, fatty fish, vegetable oils, nuts, and seeds	Protects mitochondrial membrane proteins and DNA from oxidative damage	——
Spermidine	Whole grain food, kelp, bracken, shiitake mushrooms	Cell growth, proliferation, differentiation, and antioxidants	——
Royal jelly	Bee	Regulate blood pressure and blood lipids, and improve sleep	——
Omega 3 essential fatty acids	Fish with high-fat content include salmon, herring, etc.	Maintaining cell membranes, anti-inflammatory, improving eye health, anti-anxiety, and depression	0.2× to 0.5× amount of omega 6 EFA
Omega 6 essential fatty acids	Corn, soybeans, nuts, beef, poultry, eggs, the Various common seed oils	Maintenance of cell membranes and regulation of metabolism	3–6% of total calories, or 6–12 g
Vitamin A	Animal liver, fish, eggs, etc.; carrots, and other dark-colored fruits and vegetables	Maintains the lining of the vision, skin, intestines, lungs, and urinary tract, helps protect against infection	700 μg women, 900 μg men, 1300 μg for pregnant
Vitamin B1	Grains, legumes, yeast, animal liver, eggs, vegetables, and fruits	Reduces peripheral neuropathic pain and improves skin health	1.1 mg women, 1.2 mg men
Vitamin B6	Meat, milk, egg yolk, fish, spinach, and beans	Metabolism of amino acids and fatty acids promotes epithelial cell growth and nerve function formation	1.3 mg
Vitamin B12	Animal offal, meat, shellfish, eggs, fermented foods, milk	Involved in fatty acids and amino acid metabolism, myelin synthesis	2.4 μg
Vitamin D	Exogenous sources such as fish, liver, and eggs; endogenous sources are synthesized in the skin by ultra-violet radiation	Promotes the absorption of calcium and phosphorus by the mucous membrane cells of the small intestine and the skin’s metabolism	15 μg 19–70 years women and men 20 μg women and men older than 70
Vitamin C	Fresh vegetables such as spinach, peppers, etc., and fresh fruits such as oranges, kiwi, etc.	Essential cofactor for collagen, L-carnitine, and neuro-transmitter biosynthesis, for healing wounds and burns, anti-oxidant	75 mg women, 90 mg men, 35 mg extra for smokers
Vitamin E	Nuts and seeds include almonds, hazelnuts, vegetables, egg yolk, and legumes	Anti-oxidation to prevent premature aging of skin cells	15 mg
Zinc	Shellfish, especially oysters, fish, fresh vegetables, wheat germ, whole grains, and nuts	Helps in the healing of burns and wounds and is a crucial element for growth needs and promoting good organ development	8 mg women, 11 mg men
Selenium	Meats, seafood, and cereals (dependent on the selenium content of the soil where the grain was grown)	Antioxidant properties protect the body from free radicals and carcinogens and protect the retina from damage	55 μg
Magnesium	Beans, nuts, grains, seafood, green leafy vegetables, and fruits	Facilitates nerve and muscle function, activates enzymes	310 mg women, 400 mg men
Anthocyanin	Black goji berries, black sesame seeds, blueberry, mulberry	A powerful antioxidant enhances blood vessel elasticity, improves the circulatory system and skin smoothness, inhibits inflammation	0 mg/kg∼2.5 mg/kg
Curcumin	Ginger, curry, mustard, carrot, tomato, pepper	Antioxidant inhibits corneal neo-vascularization caused by hypoxia and inflammation	——
Lutein & Zeaxanthin	Orange juice, honeydew melon, kiwi, wheat, corn, egg yolk	Anti-oxidation protects the retinal mac -ula and prevents cataracts	——
Ginkgo biloba	Ginkgo biloba tree	Anti-oxidation, promotes blood circulation, and scavenges free radicals in the body	——
Green tea	Tea	Antioxidant and anti-inflammatory	——

## 2 Oxidative stress in dry eye disease

Reactive oxygen species (ROS) are natural byproducts of normal oxidative metabolism, often perceived as detrimental due to their potential to harm biological macromolecules. The body’s antioxidant mechanisms, which include enzymatic systems such as superoxide dismutase (SOD), catalase, and glutathione peroxidase, along with non-enzymatic systems like reduced glutathione and vitamins A, C, and E, play critical roles in mitigating ROS damage (Arslan et al., 2023). Environmental stressors—including ultraviolet radiation, air pollution, and ozone, as well as physiological factors such as hormonal fluctuations, aging, exposure to pesticides, microbial antigens, and high-sugar diets can elevate ROS levels on the ocular surface, triggering oxidative stress (Honisch et al., 2023). Increased ROS levels trigger the upregulation of various antioxidant proproteins/enzymes, initiating a negative feedback mechanism to modulate stress signaling or ROS concentrations. Maintaining sufficient antioxidant capacity is vital to prevent excessive stress signals when ROS levels exceed certain thresholds. Overproduction of ROS and impaired antioxidant defenses can lead to oxidative stress, which may promote an inflammatory response. This inflammatory response on the ocular surface can lead to tissue injury, often marked by apoptosis of conjunctival goblet cells, thereby destabilizing the tear film and increasing tear osmolarity. Subsequently, this triggers further ROS production and reactivates the inflammatory cycle, exacerbating apoptosis in corneal and conjunctival epithelial cells—a process that perpetuates a vicious circle causing the progression of DED (Dammak et al., 2023). Given the rising awareness of health, the public is increasingly interested in dietary approaches to health management. Numerous studies are exploring whether nutritional supplements with antioxidant properties can ameliorate oxidative damage and restore tear film function, thereby offering therapeutic benefits in reducing ROS and managing DED.

A significant link exists between oxidative stress, systemic diseases, and DED. Conditions such as diabetes mellitus, multiple sclerosis (MS), and systemic lupus erythematosus have been associated with oxidative stress, potentially exacerbating DED (Dogru et al., 2018; Bustamante-Arias et al., 2022). Specifically, in diabetes, elevated glucose levels increase ROS production via the PI3K/AKT signaling pathway, aggravating oxidative stress in the ocular surface, damaging the epithelium cells, and affecting tear film stability, thereby leading to DED (Lu et al., 2018; Chen et al., 2022). Additionally, diabetes-related nerve injury may decrease ocular surface sensitivity and tear secretion, further aggravating DED symptoms (Trindade et al., 2021). Oxidative stress is also involved in the pathogenesis of MS, where the demyelination of nerve cells in the central nervous system could be triggered by ROS-mediated immune responses to the central nervous system (Lassmann et al., 2012; Ohl et al., 2016). Patients with MS may suffer from nerve injury affecting the ocular muscles, including those controlling blinking, potentially decreasing blinking frequency and aggravating tear evaporation, thereby triggering or worsening DED (Kumaran et al., 2000). Moreover, individuals with MS might be more developed to DED due to other complications, like autonomic dysfunction (Kıranatlı et al., 2024). The administration of antioxidants to mitigate oxidative stress and protect cells shows promise in managing DED and its associated systemic disorders (Li et al., 2022a).

## 3 Literature search methodology

The primary goal of this review was to provide a detailed overview of nutrients beneficial for DED management and to assess potential health risks associated with their use. An extensive literature search was conducted up to January 2024 across databases such as PubMed, Scopus, and Web of Science. Search terms included “dry eye disease,” “lacrimal gland,” “ocular surface health,” “ocular surface diseases,” “nutrients,” “nutritional supplements,” “dietary supplements,” “oxidative stress,” “anti-inflammatory,” “antioxidant,” “intervention treatment,” “clinical studies” and among others. This was supplemented by a thorough examination of references in key articles to ensure comprehensive coverage of relevant nutrients. Our findings preliminary confirmed ten essential nutrients that show promise for DED treatment: essential fatty acids, vitamins, trace elements, phytochemicals, L-carnitine, lactoferrin, probiotics, coenzyme Q10, spermidine, and royal jelly. Subsequently, these ten categories of nutrients were applied with “definition,” “source,” “biological function,” “use,” “mechanism of action for dry eye benefit,” “adverse effects,” “health risks,” and other related terms were then searched to ensure the completeness and coherence of the review.

## 4 Impact of nutrients on the ocular surface and potential risks

### 4.1 L-carnitine

#### 4.1.1 Definitions, biological functions, and areas of application

L-carnitine, a natural amino acid found primarily in animal-based foods, plays a multifaceted role in cellular metabolism. It regulates mitochondrial activity through modulation of transcription factors and is a crucial cofactor in the β-oxidation of fatty acids, which facilitates the cellular energy production process (Iacobazzi et al., 2013). L-carnitine also impacts lipid metabolism by influencing various transcription factors related to lipolysis and adipogenesis, reducing lipid synthesis and deposition (Förster et al., 2021). Moreover, it exhibits antioxidant properties (Sahebnasagh et al., 2022), balancing hypertonicity-induced imbalances between oxygenase and antioxidant enzymes, reducing ROS production, inhibiting lipid peroxidation, preventing oxidative DNA damage, and suppressing the production of heme oxygenase-1 and cyclooxygenase-2. Commonly used as a dietary supplement, L-carnitine is acclaimed for its benefits in boosting metabolic energy, aiding in weight loss (Talenezhad et al., 2020), and improving cardiovascular (Nakajima et al., 2024) and cognitive functions (Zhao et al., 2023).

#### 4.1.2 Mechanism of action in dry eye disease

Lucius et al. (2023) demonstrated that L-carnitine, at concentrations of 1–3 mmol/L, effectively controls DED by inhibiting the activation of the transient receptor potential vanilloid subtype 1 (TRPV1) induced by hyperosmolarity. This occurs through the blockade of calcium ion (Ca^2^⁺) influx and capsaicin-induced cell volume contraction. Notably, they observed that 1 mmol/L of L-carnitine was more effective than 3 mmol/L, suggesting that higher concentrations may exert nonselective cytotoxic effects, leading to increased Ca^2^⁺ influx and less reduction in cell volumetric contraction (Lucius et al., 2023). Further investigations by López-Cano et al. (2021) on human corneal epithelial cells (CECs) under varying osmolarities (350–500 mOsm/L) indicated that L-carnitine (50 mmol/L) and taurine at an unspecified concentration exhibited higher inhibitory activity against Tumor Necrosis Factor α (TNF-α). 

#### 4.1.3 Advantages associated with the condition of dry eye disease

Recent research has uncovered dissimilarities between DED patients and controls by examining carnitine levels in the tears of DED patients, indicating a possible link between the onset of DED and insufficient carnitine in the tear film. Some experts suppose that L-carnitine can be a metabolic biomarker for ocular diseases (Theodoridis et al., 2022). Khanna et al. (2022) utilized metabolomics to validate the role of L-carnitine in the pathogenesis of DED. They hold that L-carnitine can prevent damage to the ocular surface by regulating tear film osmolarity. Studies by Pescosolido et al. (2009); Yamaga et al. (2021) further confirmed its effectiveness in alleviating symptoms in DED patients. Ma et al. (2021) formulated a levocarnitine thermosensitive *in situ* gel (LCTG) to evaluate its efficacy in treating DED in animal models. The study demonstrated that LCTG significantly increased tear secretion, promoted the repair of CECs, as well as downregulated the expression levels of matrix metalloproteinase-3 (MMP-3) and MMP-9. In addition, the authors reported minimal stimulation and highly effective treatment of DED by LCTG in animal models. They concluded that the overall therapeutic effect of LCTG was superior to that of conventional L-carnitine solution (Ma et al., 2021).

#### 4.1.4 Possible side effects and potential health risks

While L-carnitine is recognized for its energy-enhancing properties and potential to decrease appetite, prolonged use might lead to reduced nutrient intake, potentially resulting in malnutrition and decreased physical function.

### 4.2 Lactoferrin

#### 4.2.1 Definitions, biological functions, and areas of application

Lactoferrin (LF) is a glycoprotein that mainly consists in mammalian milk and exhibits a wide range of biological functions. It plays a vital role in regulating iron metabolism by chelating iron, thereby inhibiting microbial growth through iron deficiency and interacting with lipopolysaccharide (LPS) to develop bactericidal effects (Zarzosa-Moreno et al., 2020). LF also enhances the host cell’s antiviral defenses, blocking viral replication (Ikeda et al., 2000). Additionally, it modulates inflammatory responses by reducing key cytokines such as TNF-α and interleukin 1β (IL-1β) (Asaad and Mostafa, 2022), and acts as a free radical scavenger, reducing oxidative stress and protecting cellular integrity (Ibuki et al., 2020). Due to its antibacterial, anti-infective, and immunomodulatory characteristics, LF has been incorporated into infant formula (Colombo et al., 2023) and utilized in adjunct anti-infective treatment (Jiang et al., 2014).

#### 4.2.2 Mechanism of action in dry eye disease

LF’s antioxidant properties on the ocular surface, where it chelates free iron and prevents the formation of harmful hydroxyl radicals, thus protecting CECs from oxidative stress (Pastori et al., 2015). A Randomized controlled trial (RCT) has demonstrated that LF supplementation enhances the integrity of the tear film, improves the morphology of conjunctival epithelial cells, and increases the lipid layer thickness, contributing to overall tear stability (Dogru et al., 2007). Moreover, as an antioxidant, it has demonstrated inhibitory effects on cytokine production, and it effectively attenuates the hyperinflammatory response of pathogens (Berthon et al., 2022). Additionally, LF can suppress excessive inflammation by inhibiting complement activation and reducing inflammatory mediators (Samuelsen et al., 2004).

#### 4.2.3 Advantages associated with the condition of dry eye disease

The concentration of LF in the tears of patients with DED is low, and there is a belief that LF concentration can serve as a biomarker for diagnosing DED. This belief is supported by Ponzini et al. (2020)’s meta-analysis. Innovative diagnostic technologies, such as the use of nanophotonic metasurfaces (Ye et al., 2024) and fluorescence polarization-based aptasensors (Zhang et al., 2023b), have been developed to detect LF in tear fluid, offering new approaches for accurate DED diagnosis and classification.

LF’s therapeutic effects include mitigating CECs damage and promoting cellular repair, as demonstrated in animal models of DED (Pattamatta et al., 2013; Regueiro et al., 2023). Connell et al. (2021) also observed that LF can increase tear secretion, inhibit the expression of inflammatory factors, and induce a short-lasting effect on tear secretion by modulating the gut microbiota to stimulate the production of short-chain FAs in a mouse model of DED. However, it should be noted that in this experiment, vancomycin, a type of antibiotic, caused LF ineffective in treating DED by impacting the gut flora (Connell et al., 2021). With the growing research on the efficacy of LF in DED, many researchers have developed LF-loaded liposomes using nanomaterials. This addresses the poor aqueous stability of LF and its high excretion through the nasolacrimal duct, which can affect its therapeutic efficacy. López-Machado et al. (2021) have developed liposomes with LF that exhibit good anti-inflammatory properties, effectively alleviating discomfort without irritation.

#### 4.2.4 Possible side effects and potential health risks

According to the data, LF demonstrates minimal side effects, particularly in bovine LF. While LF is generally well-tolerated, some side effects such as temporary fecal loosening have been noted, particularly with oral administration, necessitating careful monitoring during extended use (Dogru et al., 2007). Long-term administration must be managed to avoid issues such as excessive iron absorption, which could lead to digestive disturbances and potentially interfere with the absorption of other vital nutrients.

### 4.3 Probiotics

#### 4.3.1 Definitions, biological functions, and areas of application

Probiotics are live microorganisms that offer health benefits. These include the ability to suppress or eliminate harmful bacteria, balance the intestinal microecology, promote digestion and nutrient absorption, improve the intestinal environment, stimulate immune cell function, and strengthen the body’s immune response (da Silva et al., 2024). Probiotics exhibit antibacterial and anti-inflammatory properties and are commonly used in fermented dairy products, beverages, and other functional foods. They are also employed in managing intestinal disorders and as a supplemental therapy to enhance immune functions (Abouelela and Helmy, 2024).

#### 4.3.2 Mechanism of action in dry eye disease

Recent studies have advanced our understanding of the ocular surface microbiota, identifying key bacterial groups such as *Firmicutes* (such as *Staphylococcus* and *Streptococcus*), *Actinomycetes* (specifically *Corynebacterium*), and *Proteobacteria* (including *Acinetobacter* and *Pseudomonas*). These groups are crucial for pathogen defense and play significant roles in regulating the ocular immune response, thus maintaining ocular health (Li et al., 2020).

The Gut-Eye Axis concept suggests intestinal dysbiosis may significantly impact the onset and progression of eye diseases, including autoimmune DED. Patients with autoimmune DED often experience damage to ocular surface tissues such as the cornea, conjunctival goblet cells, and lacrimal glands. This damage is frequently associated with alterations in gut microbial diversity and abundance (Bai et al., 2023), which can trigger systemic inflammation and contribute to a range of ocular disorders, including age-related macular degeneration, uveitis, diabetic retinopathy, and glaucoma (Bai et al., 2023; Campagnoli et al., 2023).

According to Donabedian et al. (2022), autoimmune uveitis induced by gut flora may be attributed to four factors. Firstly, the gut flora regulates the levels of microbial metabolites, such as butyric acid and short-chain FAs, which have anti-inflammatory properties. Secondly, Imbalances caused by gut dysbiosis can disturb immune homeostasis, affecting various body organs. Thirdly, an imbalance between helper T-cells 17 (Th17) and regulatory T-cells (Treg) can lead to excessive IL-17 production, exacerbating inflammation. Lastly, microbial recognition in the gut can activate autoantigenic T cells in the uvea, contributing to autoimmune reactions (Donabedian et al., 2022). These four factors are closely associated with the ocular manifestations of systemic autoimmune disease, and it is highly probable that uveitis directly contributes to the development of DED. Schaefer et al. (2022) also discovered that the gut microbiota can influence the health of the ocular surface by impacting CD4^+^ FOXP3+ Tregs in the lymph nodes of the eye.

Studies have also shown that imbalances in gut microbiota can amplify the inflammatory response to LPS, a component of gram-negative bacteria, aggravating DED (Bertani and Ruiz, 2018; Wang et al., 2019). Such disruptions have been associated with worsened DED symptoms, decreased corneal barrier function, and reduced epithelial cell density, indicating a negative correlation between microbial diversity and DED severity (Mendez et al., 2020).

Research by Qi et al. (2021) highlighted differences in gut microbiota between individuals with autoimmune disease-mediated DED and those with non-autoimmune DED. Findings included variations in bacterial populations like *Corynebacterium*, *Streptococcus*, and *Prevotella*, correlating with DED severity. Additionally, a separate study reported an elevated presence of *Veillonella* in individuals with DED compared to the gut microbiota of the Sjögren syndrome and healthy population. Meanwhile, the *Subdoligranulum* was significantly reduced in patients with DED (Moon et al., 2020a). Additionally, research has substantiated the significance of *Prevotella* in the modulation of tear secretion among individuals suffering from DED and demonstrated a positive correlation between the severity of DED and the presence of *Prevotella* (Zhang et al., 2023a).

Research has indicated that dietary choices, particularly high-fat diets, significantly influence the gut microbiota, exacerbating DED symptoms through mechanisms such as decreased tear secretion, increased oxidative stress, and heightened apoptosis (Wu et al., 2020). Furthermore, research by Zhang et al. (2022a) observed that a high-fat diet led to excessive growth of intestinal *Deferribacterota* in a mouse model of desiccation syndrome, closely correlating with increased severity of DED.

#### 4.3.3 Advantages associated with the condition of dry eye disease

Recent research underscores the potential of optimizing gut microbiota to treat DED effectively. Tavakoli et al. (2022) highlighted that both probiotic and prebiotic therapies have shown promising results in treating DED. Specific strains such as *Bifidobacterium lactis* and *Bifidobacterium bifidum* have been documented to improve tear secretion and BUT (Chisari et al., 2017b). Additionally, *Saccharomyces boulardii* MUCL 53837 and *Enterococcus faecium* LMG S-28935 have been effective in alleviating subjective symptoms of DED (Chisari et al., 2017a). Further research has demonstrated the efficacy of a complex of five probiotics in enhancing tear production in both autoimmune and dry-stress DED mouse models, suggesting a broader therapeutic potential across different DED types (Moon et al., 2020b; Choi et al., 2020). Another study revealed that an oral formulation of *Bifidobacterium* and *Lactobacillus plantarum* not only increased tear secretion but also repaired CECs and reduced inflammatory markers like TNF-α and IL-1β (Yun et al., 2021).

In an RCT with 60 participants, a group receiving a probiotic preparation of *Enterococcus faecalis* and *S. boulardii* along with routine eye drops showed significant improvement in DED symptoms compared to the control group, which received only eye drops (Chisari et al., 2017a). Another RCT investigating a combination of *Lactobacillus*, *Bifidobacterium*, and *Streptococcus* with NutriKaneD confirmed its effectiveness in increasing tear secretion and reducing discomfort (Tavakoli et al., 2022). Building upon these studies, Lee et al. (2023) conducted a detailed investigation on the effects of *Lactobacillus fermentum* HY7302 in a benzalkonium chloride-induced mouse model of DED and found that it reduced corneal fluorescein scores, increased tear secretion, and repaired CECs. It also decreased oxidative stress and inflammatory cytokine production in the DED model. Concerning the utilization of probiotics, Heydari et al. (2023) evaluated the effectiveness and safety of administering *Latilactobacillus sakei* both orally and topically in DED patients. The study found that while both methods were safe, topical administration was more effective at reducing inflammation (inhibition of inflammatory factors such as IL-6, TNF-α, and Interferon-gamma) and improving tear stability (Heydari et al., 2023).

Aside from the orally administered probiotic preparations, there is a novel medical therapy called intestinal flora transplantation (FMT) that aims to reinstate the equilibrium of intestinal flora in patients through the transfer of fecal flora from healthy individuals to the patient’s intestines. This therapy has already proven effective in treating various conditions, including *Clostridium difficile* infections (Hui et al., 2019), Crohn’s disease (Fehily et al., 2021), irritable bowel syndrome (El-Salhy et al., 2021), and psychiatric disorders (Pascale et al., 2020). Initial studies have confirmed its safety and efficacy in a small cohort of patients with immune-mediated DED (Watane et al., 2022).

#### 4.3.4 Possible side effects and potential health risks

While probiotics are generally safe, they can cause adverse reactions, including bacterial and fungal sepsis, immune system hyperstimulation, microbial resistance, and gastrointestinal issues, especially in vulnerable populations like premature infants and immunocompromised individuals (Sotoudegan et al., 2019). It is critical to monitor patients closely when using probiotics in clinical settings. Furthermore, challenges remain regarding the colonization capacity of probiotic strains and the standardization of probiotic preparations (Donabedian et al., 2022).

### 4.4 Coenzyme Q10

#### 4.4.1 Definitions, biological functions, and areas of application

Coenzyme Q10, also known as ubiquinone, is a lipid-soluble compound found predominantly in mitochondria and cell membranes. It is critical for cellular functions (Hargreaves et al., 2020), particularly in energy production through adenosine triphosphate synthesis (Aussel et al., 2014), acting as an antioxidant to combat free radicals (Bentinger et al., 2007), and inhibiting lipid peroxidation to protect cell membranes from oxidative damage (Zhang et al., 2013). Clinically, CoQ10 is utilized in the management of heart failure and myocarditis, owing to its ability to preserve the morphology and structure of mitochondria in ischemic cardiomyocytes (Alarcón-Vieco et al., 2023). Moreover, it is also used in the treatment of antioxidants (Samimi et al., 2024) and maintenance of muscle function (Talebi et al., 2024).

#### 4.4.2 Mechanism of action in dry eye disease

A causal relationship exists between oxidative stress and mitochondrial dysfunction, as the copious amount of free radicals produced by oxidative stress can cause damage to mitochondrial complex I. Conversely, suppressing mitochondrial complex I results in an escalation in the generation of free radicals. It is, therefore, imperative to safeguard the function of mitochondria and prevent any potential dysfunction to effectively treat diseases such as DED (López-Lluch, 2021). CoQ10 also inhibits apoptosis by specifically safeguarding mitochondrial DNA and inhibiting mitochondrial depolarization. In cases of oxidative stress, it scavenges oxygen free radicals and keeps mitochondrial DNA deletion mutations in check, maintaining the whole respiratory chain. Additionally, CoQ10 binds to the mitochondrial permeability transition pore, preventing mitochondrial depolarization and cytochrome C release to lower cysteine protease-3 activation and reduce apoptosis (Bentinger et al., 2007; Hseu et al., 2018; Fatima et al., 2021).

#### 4.4.3 Advantages associated with the condition of dry eye disease

Research indicates that CoQ10 significantly improves lacrimal gland function and reduces inflammation due to its antioxidant properties. Studies using animal models also have demonstrated its effectiveness in mitigating mitochondrial damage linked to oxidative stress in the lacrimal gland (Uchino et al., 2012). Clinical trials using combinations of CoQ10 with cross-linked hyaluronic acid (XL-HA) have shown enhanced treatment outcomes in DED patients, attributed to CoQ10’s antioxidant effects and HA’s inherent water retention and viscoelasticity properties, which are essential for repairing ocular tissues (Postorino et al., 2018; Posarelli et al., 2019). In another study, researchers concluded that including CoQ10 in treatment reduced all cytokine levels and a notable elevation in total antioxidant status levels. The efficacy of CoQ10 in enhancing protection against oxidative damage and safeguarding the lacrimal gland was established through histopathological and tissue cytokine level analyses (Yakin et al., 2017). Serrano-Morales et al. (2022) conducted a prospective study using a double-blind approach to assess the effectiveness of a combination supplement containing XL-HA, CoQ10, and vitamin E in women experiencing menopausal DED symptoms while undergoing antidepressant treatment. The study’s results indicated that patients who received the intervention experienced improvements in DED symptoms, as well as increased tear film stability (measured by the BUT test) and higher tear production (measured by the Schirmer test). It is worth noting that HA exhibits favorable lubricating properties, particularly when cross-linked, as it forms a liquid matrix on the ocular surface. Moreover, CoQ10 and vitamin E in this combination supplement act as antioxidants, improving ocular repair (Serrano-Morales et al., 2022). Additionally, Tredici et al. (2020) demonstrated that applying this complex can protect the ocular surface of professional swimmers frequently exposed to chlorinated water for extended periods.

#### 4.4.4 Possible side effects and potential health risks

When evaluating potential drug interactions, it is imperative to consider the impact of CoQ10 on warfarin metabolism. Due to its structural similarities with vitamin K, CoQ10 may selectively interact with cytochrome P450 enzymes, reducing response to warfarin (Sharma et al., 2016). This could introduce challenges for patients with lifelong anticoagulation needs, such as those with heart failure and atrial fibrillation (Ayers et al., 2018). Additionally, CoQ10’s strong antioxidant properties might reduce the effectiveness of pro-oxidant chemotherapies (Yasueda et al., 2016) and could interact with antihypertensive drugs to cause an excessive drop in blood pressure, necessitating careful monitoring and dose adjustments (Zhao et al., 2022).

### 4.5 Spermidine

#### 4.5.1 Definitions, biological functions, and areas of application

Spermidine is a naturally occurring polyamine found in all living organisms, playing essential roles in cellular metabolism (Hofer et al., 2021), including cell proliferation and division (Luo et al., 2023). It is critical for maintaining DNA stability and synthesis (Wang et al., 2018) and supports processes such as apoptosis and autophagy, crucial for removing damaged cells and maintaining cellular health (Hofer et al., 2021). Due to its extensive biological roles, spermidine is used to reduce inflammatory responses (Mao et al., 2023), improve cardiovascular health (Wu et al., 2022), and enhance cognitive functions (Pekar et al., 2021).

#### 4.5.2 Mechanism of action in dry eye disease

Spermidine helps regulate the immune system’s balance, crucial in conditions like DED, where inflammation plays a key role. M1/M2 macrophage polarization and the Treg/Th17 cell balance, are processes integral to modulating inflammation (Shapouri-Moghaddam et al., 2018; Zhang et al., 2021). DED is associated with an imbalance in Th17/Treg cells, leading to increased Th17 polarization and secretion of pro-inflammatory cytokines like IL-17, causing epithelial damage (Ratay et al., 2017). As we all know, Macrophage-induced inflammation promotes the differentiation of CD4 T cells into Th17 cells, which inhibits Treg cell function. Spermidine regulates immune cell number and function by inducing macrophage polarization and CD4 T cell differentiation in an autophagy-dependent manner (Liu et al., 2020; Carriche et al., 2021). It also acts as an antioxidant and specific calcium chelator, safeguarding cells from oxidative stress-induced calcium overload and preventing apoptosis when exposed to oxidative or endoplasmic reticulum stress (Kim et al., 2021). Meanwhile, it is primarily found in the brain and retina and is an endogenous scavenger for free radicals. It also exhibits potential inhibitory effects against ROS, as observed in previous studies (Ha et al., 1998).

#### 4.5.3 Advantages associated with the condition of dry eye disease

Spermidine has shown potential in inhibiting the production of pro-inflammatory cytokines in microglia activated by LPS and protecting fibroblasts from oxidative damage induced by hydrogen peroxide (Horrocks and Yeo, 1999; Rider et al., 2007). These properties contribute to enhanced tear film stability and reduced ocular surface inflammation in DED models. In mouse studies, spermidine treatment resulted in decreased IL-17 levels in the corneas and lacrimal glands, stabilizing the tear film and mitigating ocular surface inflammation (Lee et al., 2021).

#### 4.5.4 Possible side effects and potential health risks

While spermidine offers therapeutic benefits, its use in treating ocular conditions must be approached with caution due to potential cytotoxic effects. These include the risk of excessive proliferation and migration of retinal pigment epithelium cells, which may lead to complications such as hyperproliferative retinopathy (Han et al., 2022).

### 4.6 Royal jelly

#### 4.6.1 Definitions, biological functions, and areas of application

Royal Jelly (RJ) is a creamy secretion produced by the glands of worker bees (Wang et al., 2023), known for its antibacterial, antifungal, anti-inflammatory, and antioxidant properties (Ghadimi-Garjan et al., 2023). It supports cell renewal, wound healing, and blood pressure and lipid regulation (Zamami et al., 2008). Given its nutrient richness and biofunctional diversity, RJ is widely used as a dietary supplement and for antibacterial and anti-inflammatory treatments (Baptista et al., 2023).

#### 4.6.2 Mechanism of action in dry eye disease

RJ exhibits natural antioxidative characteristics, significantly reducing ROS and nitric oxide production in macrophages and boosting activities of antioxidant enzymes like SOD and glutathione (Gu et al., 2018). Major Royal Jelly Protein 2, a key RJ component, diminishes oxidative stress and inhibits apoptosis in microbial cells, enhancing cell survival under oxidative conditions (Abu-Serie and Habashy, 2019). Additionally, RJ has shown efficacy in reducing oxidative stress in diabetic animal models by enhancing antioxidant enzyme activities and reducing oxidative damage markers (malondialdehyde levels) (Ghanbari et al., 2016).

RJ’s anti-inflammatory effects are mediated through its fatty acid analogs, including 10-hydroxy-2-decanoic acid, 10-hydroxy decanoic acid, and sebacic acid, which effectively inhibit the release of pro-inflammatory cytokines and other inflammatory mediators (Yang et al., 2018b). These compounds influence critical inflammatory pathways, such as mitogen-activated protein kinases and NF-κB signaling pathways, and promote the production of anti-inflammatory cytokines IL-1ra (You et al., 2020).

#### 4.6.3 Advantages associated with the condition of dry eye disease

RJ has been used in conventional ophthalmic remedies like honey eye drops for corneal wound healing (Azmi et al., 2021) and propolis for protecting retinal neurons (Abd Rashid et al., 2022). Studies have demonstrated that RJ enhances tear secretion by promoting Ca^2+^ mobilization and conserving ATP in lacrimal glands through muscarinic signaling pathways (Inoue et al., 2017). Yamaga et al. (2021) investigated the impact of various bioactive components in RJ on tear production. To determine the critical component responsible for increasing tear production, they utilized a mouse model of stress DED. Their research discovered that a combination of three FAs and acetylcholine played a pivotal role in enhancing tear production (Yamaga et al., 2021). An extensive study comparing various bee products (including raw honey, propolis, RJ, pollen, and bee larvae) found RJ most effective in restoring tear secretion and maintaining mitochondrial health in lacrimal glands (Imada et al., 2014).

A clinical trial also confirmed that oral RJ supplementation significantly improved tear secretion in DED patients without adverse effects (Inoue et al., 2017). Further, systematic reviews have validated RJ’s therapeutic impact on improving DED symptoms (Prinz et al., 2023).

#### 4.6.4 Possible side effects and potential health risks

Despite its benefits, RJ may cause allergic reactions, including skin rashes and pruritus, particularly in sensitive individuals (Li et al., 2021). Due to its estrogenic properties, RJ is not recommended for children under 10 years and pregnant women (Ishida et al., 2022). However, no significant adverse reactions have been reported in clinical studies.

### 4.7 Nutrients are currently recognized as beneficial for dry eye disease

Nutrient therapy is a significant component in the holistic management of DED, supplementing medical treatments. Various studies have highlighted the therapeutic benefits of diverse nutrient compositions such as ω-3FAs (Roncone et al., 2010; Rosenberg and Asbell, 2010), vitamins (Gorimanipalli et al., 2023), trace elements, and phytochemicals (Pellegrini et al., 2020) for treating DED. Given that the efficacy of these nutrients in treating DED has been thoroughly explored through reviews and meta-analyses, this review will focus on elucidating their mechanisms of action and potential health risks. This approach aims to underscore the importance of ensuring the effectiveness of nutritional supplements in alleviating DED symptoms while ensuring their safety.

#### 4.7.1 Essential fatty acids

##### 4.7.1.1 Definitions, biological functions, and areas of application

Essential fatty acids (EFAs), including linoleic acid and α-linolenic acid from the ω-6 and ω-3 series respectively, are crucial to the structure and function of human cell membranes. They play vital roles in maintaining membrane integrity and fluidity, which is essential for cell viability and function (Mason et al., 2016). EFAs are also indispensable for growth and development, particularly in brain and nervous system development (Lin et al., 2024). The cognitive benefits of α-linolenic acid and the role of Docosahexaenoic Acid (DHA) in visual maintenance are well-documented (Sala-Vila et al., 2009; Horrocks and Yeo, 1999). Additionally, EFAs contribute to lowering blood cholesterol and triglyceride levels, thereby reducing the risk of cardiovascular disease (Sherratt et al., 2024). Their anti-inflammatory properties are beneficial in alleviating symptoms of inflammatory diseases such as DED (Lorente-Cebrián et al., 2015). The widespread recognition of the health benefits associated with EFAs has led to the availability of various health foods and supplements containing these compounds.

##### 4.7.1.2 Mechanism of action in dry eye disease

EFAs, specifically ω-3FAs, are critical in preventing and managing DED. Their mechanism of action involves several facets: initially, they inhibit the production and release of inflammatory mediators such as prostaglandins, leukotrienes, and tumor necrosis factor, which are pivotal in the pathogenesis of DED (Sheppard et al., 2013). By diminishing the activity of these inflammatory factors, ω-3FAs mitigate the inflammatory response in ocular tissues, thereby alleviating symptoms of DED. Moreover, ω-3FAs positively influence eye cell repair and regeneration, promoting the healing of damaged corneal epithelial and lacrimal gland cells, thus aiding in tissue integrity and function restoration (Roncone et al., 2010).

##### 4.7.1.3 Possible side effects and potential health risks

Short-term gastrointestinal disturbances are the most reported adverse reactions in the EFAs correlation reports. Of the studies that evaluated the administration of ω-3FAs as complementary agents versus placebo and reported adverse reactions, most disclosed gastrointestinal disturbances, including diarrhea, in the ω-3FAs group (Bhargava et al., 2016; Deinema et al., 2017; Dry Eye Assessment and Management Study Research Group et al., 2018). While these side effects were relatively rare, and there was not a significant observable difference between ω-3FAs and placebo, it is imperative to consider the safety of systemic replenishment of ω-3FAs as a crucial aspect. Patients with atrial fibrillation, liver disease, or bleeding disorders are preferably advised against ω-3FAs supplements (Jones et al., 2017). Although dietary therapy is generally deemed safe for healthy adults, high doses of ω-3FAs supplementation (>2,000 mg/d) have been linked to a slight increase in bleeding risk in specific populations (Buckley et al., 2004). At the same time, exceeding a certain threshold of ω-3FAs concentration may enhance the risk of bleeding and arrhythmias (Kapoor et al., 2021). Further investigations are needed to confirm the safety of ω-3FAs. The composition, dose, course, and application method of EFAs preparations (diet, capsules, eye drops) are essential considerations for effectively treating DED.

#### 4.7.2 Vitamins

##### 4.7.2.1 Definitions, biological functions, and areas of application

Vitamins are essential organic compounds that serve as crucial micronutrients for maintaining vital bodily functions and overall health. They significantly influence growth, development, and physiological processes. Vitamin A is vital for vision and immune system support (Dewett et al., 2021), while Vitamin D enhances bone health and facilitates calcium absorption (Rizzoli et al., 2014). Vitamins C and E are known for their antioxidant properties, which help combat oxidative stress (Myhrstad and Wolk, 2023). Typically used to meet the nutritional needs of various populations, including children, pregnant women, and the elderly, vitamins also play a role in preventing and treating deficiencies.

##### 4.7.2.2 Mechanism of action in dry eye disease

Vitamins are integral in modulating immune responses within the body, including the ocular system. For example, Vitamin D receptors research found within the human eye suggest that Vitamin D plays a significant role in eye cell functions and may reduce ocular surface inflammation associated with DED, thereby improving symptoms (Caban and Lewandowska, 2022). Similarly, Vitamin D supplementation has shown to improve serum Vitamin levels, enhancing ocular surface health and tear quality (Yang et al., 2018a). Vitamin A supports ocular surface repair and maintenance (Samarawickrama et al., 2015), and Vitamins C and E, both potent antioxidants, help alleviate symptoms of DED by reducing oxidative stress (Huang et al., 2016).

##### 4.7.2.3 Possible side effects and potential health risks

While vitamins are essential for health, excessive intake can lead to adverse effects (Fassier et al., 2019). High doses of Vitamin A may increase the risk of lung cancer among high-risk groups, such as smokers and asbestos workers (O’Connor et al., 2022). Furthermore, excessive Vitamin A intake has been linked to teratogenic effects and an increased risk of birth defects when consumed at high levels (over 10,000 IU/d) during pregnancy (Hunt, 1996). It can also negatively impact bone quality and increase fracture risks, such as retinoic acid can inhibit osteoblast activity, stimulate osteoclast formation, induce bone resorption, and negatively impact bone quality (Li et al., 2019). A single or short-term dose of approximately 50,000 IU can provoke toxic conditions such as vomiting, increased cerebrospinal fluid pressure, blurred vision, and impaired muscle coordination (Bendich and Langseth, 1989). Symptoms of hypervitaminosis A may typically resolve within a week of discontinuation, though long-term or irreversible effects may include cirrhosis and bone changes (Eldredge et al., 2022).

Vitamin D pre-supplementation to prevent illnesses is gaining traction among the general population. Meanwhile, it is essential to note that administering vitamin D without proper scientific guidance may lead to elevated serum concentrations of 25-hydroxyvitamin D and free 1,25-dihydroxy vitamin D, as well as an array of chronic toxic effects, such as hypercalcemia and hypercalciuria, that can result in renal calcium deposition. Employing non-hydroxylated vitamin D forms that are effectively maintained in conjunction with the body’s self-regulation of vitamin D activation may help minimize the risk of toxicity (Pludowski et al., 2018).

In a recent large-scale Mendelian randomized analysis, based on the European population cancer GWAS, the authors found that circulating vitamin E was significantly associated with an increased risk of bladder cancer (Xin et al., 2022). It has also been reported that consuming 400 IU/d of vitamin E did not prevent cancer or vascular disease and may even raise the danger of heart failure in those with underlying conditions (Lonn et al., 2005). A meta-analysis of 19 RCTs involving 135,967 subjects with daily vitamin E intake between 16.5 and 2000 IU. The authors concluded that there was a dose-response relationship between vitamin E supplementation and all-cause mortality and that high doses of vitamin E (>/ = 400 IU/d) should be avoided (Miller et al., 2005). At the same time, we must be aware that hypervitaminosis E status may cause abnormal bleeding events by suppressing the synthesis of vitamin K-derived coagulation factors (Abrol et al., 2023).

Throughout various studies, high doses of vitamin C have generally been observed to be well-tolerated. However, there have been indications that regular doses of ≥1 g over extended periods, such as months or years, may reduce bactericidal activity in leukocytes and increase the risk of stone formation caused by temporary urate excretion elevation. In addition, it is essential to consider the destructive effect of elevated levels of vitamin C on erythrocytes and the potential occurrence of hemolysis (Shilotri and Bhat, 1977; Doseděl et al., 2021).

#### 4.7.3 Trace elements

##### 4.7.3.1 Definitions, biological functions, and areas of application

Trace elements are chemical elements present in minute quantities within organisms yet are crucial for their normal physiological functions. Their biological roles are manifold. Primarily, trace elements serve as constituents of enzymes, enhancing their catalytic actions. For example, zinc is vital for protein synthesis, gene expression, and cell signaling (Kamińska et al., 2021), while copper plays a significant role in gene expression and cell differentiation (Kersey et al., 2024). Selenium is known for its antioxidant properties, helping to eliminate free radicals and protect cells from oxidative damage (Rayman, 2012). The utilization of trace element supplements is widespread for managing and preventing deficiencies in these elements, such as iron deficiency anemia and zinc inadequacy (Moradveisi et al., 2019; Sonmez Ozkarakaya et al., 2021).

##### 4.7.3.2 Mechanism of action in dry eye disease

Zinc is critical in activating numerous enzymes and cell signaling pathways (Kamińska et al., 2021). It supports cellular repair and regeneration, which is crucial for restoring functionality in damaged ocular surface cells, thus alleviating DED symptoms. It also modulates immune responses, potentially reducing inflammation and mitigating immune-mediated damage in DED (Gilbert et al., 2019). Selenium, as an antioxidant, plays a crucial role in minimizing cellular damage caused by oxidative stress, safeguarding the structure and function of the ocular surface (Higuchi, 2019).

##### 4.7.3.3 Possible side effects and potential health risks

While essential for health, it is critical to maintain optimal concentrations of trace elements. Excessive zinc can interfere with copper absorption, potentially leading to copper deficiency, which is critical for erythropoiesis and can increase the risk of anemia (Duncan et al., 2015). According to the latest study, there appears to be a strong correlation between hyperselenemia and non-alcoholic fatty liver disease (NAFLD). Wang et al. (2021) conducted a linkage analysis that examined the relationship between serum selenium concentration, alanine aminotransaminase activity, and the incidence of NAFLD in US adults. Results from this study indicate that serum selenium concentration and alanine aminotransaminase activity are nonlinearly dependent on the incidence of NAFLD. Specifically, a positive correlation was observed at serum selenium levels above 130 μg/L, while no association was observed below this level (Wang et al., 2021). Recent observational studies and RCT evidence suggest high selenium exposure may negatively impact cardio metabolism, especially hypertension, dyslipidemia, and type 2 diabetes (Vinceti et al., 2018; Zhang et al., 2022b; Li et al., 2023).

#### 4.7.4 Phytochemicals

##### 4.7.4.1 Definitions, biological functions, and areas of application

Phytochemicals are natural compounds found in plants that enhance plant defenses against pathogens (Zaynab et al., 2018) and have various beneficial effects on human health. These include anti-inflammatory and antioxidant properties, boosting the immune system, and promoting cardiovascular health (Choudhary et al., 2024; Muscolo et al., 2024; Weerawatanakorn et al., 2024). A diet rich in phytochemicals is crucial for deriving these health benefits, as exemplified in Table 1.

##### 4.7.4.2 Mechanism of action in dry eye disease

Phytochemicals exhibit potent antioxidant properties by eliminating free radicals, thereby reducing oxidative stress and protecting ocular tissues from damage (Ozawa et al., 2015). They also possess anti-inflammatory characteristics that alleviate eye inflammation and improve the inflammatory response associated with DED (Yoon et al., 2023). Additionally, these compounds enhance microcirculation within the eyes, improving the delivery of nutrients and oxygen to ocular tissues, thus mitigating symptoms of DED (Harris et al., 2019). Among the things to keep in mind is Lutein, a carotenoid abundant in the macula, shields the eyes by absorbing and filtering detrimental blue light, safeguarding the retina, and mitigating the impact of blue light-induced damage (Widomska et al., 2023).

##### 4.7.4.3 Possible side effects and potential health risks

While many phytonutrients can aid in disease prevention due to their immune-boosting, antitumor, antioxidant, antibacterial, and cardiovascular properties, excessive dosing can have potential risks. A study suggests that long-term intake of β-carotene, retinol, and lutein nutritional supplements alone to prevent diseases, especially in patients with underlying conditions and smokers, is not recommended. Although β-carotene, retinol, and lutein supplements theoretically have beneficial health impacts, the study’s authors found, in a review of the long-term consumption of these nutrients and cancer risk, that it may lead to an increased risk of lung cancer (Satia et al., 2009). Meanwhile, Ginkgo biloba has been observed to contribute to hemodilution, inhibit thrombosis, and enhance circulation. Therefore, it is recommended that individuals at an elevated risk of bleeding, those taking specific anticoagulants and antiplatelet agents, and those undergoing surgical or other invasive operations should avoid it (Rosenblatt and Mindel, 1997). The potential impact of green tea on tear composition has garnered attention. A recent study investigated the influence of green tea on tears in individuals with good health. The researchers observed a decline in Phenol red thread readings and an elevation in tear ferning test grades following green tea consumption. Specifically, the Phenol red thread test, a conventional method for assessing tear production, indicated reduced readings, implying decreased tear output. Additionally, the tear ferning test, a diagnostic process that assesses tear quality through the examination of tear crystalline morphology, in patients with DED, tear ferning test grades up, and tears fern-like crystals are reduced or fragmented. Based on these findings, the authors postulate that green tea might compromise tear quality and advocate further investigation into this phenomenon (Masmali et al., 2019).

## 5 Conclusion and future perspectives

With the increase in the use of digital devices and the population aging, DED has become a common concern in contemporary society. Exploring treatment modalities for this condition has attracted considerable attention in the field of ophthalmology. Through an enhanced comprehension of the pathophysiological mechanisms underlying DED and the conduct of numerous clinical investigations, it has been discerned that antioxidants and alternative dietary supplements hold promise for preventing and managing DED. The present review has collated the foremost categories of nutrients known to offer advantages in managing DED, encompassing EFAs, vitamins, trace elements, and phytochemicals, along with six additional groups of emerging nutrients supported by studies demonstrating their efficacy in alleviating symptoms of DED. This study is intended to provide some perspective on the composition of the ideal nutrient for DED treatment in terms of its efficacy and safety.

This review has detailed the biological functions, benefits, and mechanisms of action of these six emerging nutrients in DED, but much future research exists that could be conducted within this context. Specifically, these are as follows: While current research on L-carnitine in DED is limited, its potential effects on energy metabolism, antioxidant capabilities, and cell signaling pathways could enhance lacrimal cell health and function. Future studies should aim to clarify the role of L-carnitine in ocular health, particularly how it affects lacrimal gland function. Future research could investigate potential dietary adjustments to enhance L-carnitine consumption, considering the elevated levels of L-carnitine found in meat and dairy items. This may offer a supplementary approach for managing DED conditions.

Although LF is known for its extensive biological functions, its specific interactions within the ocular surface microenvironment need deeper exploration. The potential of peptides derived from LF, which may have heightened biological efficacy, deserves further investigation in DED management (Yen et al., 2024).

Given the health benefits of probiotics, more clinical trials are necessary to evaluate the effects of different probiotic strains on DED.

Innovation in the administration of CoQ10, such as sustained-release ocular drops and nanoparticle systems, could significantly improve its effectiveness by enhancing absorption. This offers a promising direction for further research and development in optimizing CoQ10 therapy for ocular surface diseases.

While research on the therapeutic and preventive effects of spermidine on DED conditions is limited, it remains considerable to investigate the potential of spermidine to enhance tear film stability through its impact on the lipid layer, thereby reducing excessive tear evaporation.

It is essential to emphasize not only the importance of determining the optimal dosage of various nutrients for therapeutic purposes while avoiding potential health risks and ensuring cautious administration across the population but also to consider the mode of administration (be it capsules, tablets, or eye drops) and the synergistic therapeutic effects when combining them with other treatment modalities. For instance, the specific EPA to DHA ratio in EFAs, the bioavailability of different forms of zinc, and the potential utilization of innovative delivery systems like liposomes and nanoparticles warrant thorough investigation. These research areas aim to meet the individualized needs of DED patients, thus paving the way for more efficient, safer, and personalized treatment approaches.
